# Improving the Lifestyle of Adolescents Through Peer Education and Support in Vietnam: Protocol for a Pilot Cluster Randomized Controlled Trial

**DOI:** 10.2196/15930

**Published:** 2020-06-26

**Authors:** Hong K Tang, Ngoc-Minh Nguyen, Michael J Dibley, Trang H H D Nguyen, Ashraful Alam

**Affiliations:** 1 Department of Epidemiology Faculty of Public Health Ho Chi Minh City Vietnam; 2 The Sydney School of Public Health Faculty of Medicine and Health The University of Sydney Sydney Australia

**Keywords:** peer education, peer support, peer leader, adolescents, dietary behaviors, physical activity, Vietnam

## Abstract

**Background:**

In Ho Chi Minh City, Vietnam, recent studies found a rapid increase in overweight and obesity in adolescents. There is a need for effective health promotion interventions to support healthy diets and encourage a physically active lifestyle. This study will help fill an evidence gap on effective interventions to prevent excess weight gain in adolescents and generate new insights about peer-led education to promote healthy lifestyles.

**Objective:**

We aim to assess the feasibility and acceptability of a combined peer-led and peer support intervention among junior high school students in Ho Chi Minh City. Additionally, the efficacy of the intervention on adolescents’ dietary practices and time spent on physical activity will also be measured in this pilot study.

**Methods:**

The Peer Education and Peer Support (PEPS) project is a pilot cluster randomized controlled trial with 2 intervention and 2 control schools. The intervention consists of 4 weekly education sessions of why and how to choose healthy food and drinks and how to be more physically active. Additionally, the intervention includes a school-based and online support system to help maintain student engagement during the intervention. We will use in-depth interviews with students, peer leaders, teachers, and parents; focus group discussions with peer educators; and direct observation of the school environment and peer leaders’ interactions with the students. Acceptability and feasibility of the intervention will be assessed. We will also quantitatively assess limited efficacy by measuring changes in student’ physical activity levels and dietary behaviors.

**Results:**

We delivered the peer education intervention at the start of each school year over 3 months for all new grade 6 adolescents in the selected schools, followed by peer support and home engagement activities over 6 months until the end of the school year. There was a baseline assessment and 2 post-intervention assessments: the first immediately after the intervention to assess the short-term impact and the second at the end of the school year to assess the sustained impact on changes in adiposity, diet, and physical activity.

**Conclusions:**

The findings of this study will be used to develop a larger-scale cluster randomized controlled trial to examine the impact of a multicomponent, school- and home-based health promotion intervention. The trial will use innovative peer education methods to reduce overweight and obesity and improve dietary choices and physical activity levels in Vietnamese adolescents.

**Trial Registration:**

Australian New Zealand Clinical Trials Registry ACTRN12619000421134; https://www.anzctr.org.au/Trial/Registration/TrialReview.aspx?id=376690&isReview=true

**International Registered Report Identifier (IRRID):**

DERR1-10.2196/15930

## Introduction

### Background

Child overweight and obesity is one of the major risk factors for health in later life, including cardiovascular diseases, some cancers, and lower quality of life in general [1-3]. Overweight and obesity are estimated to cause 3.4 million deaths and account for 4% of years of life lost and 4% of disability-adjusted life years (DALYs) worldwide [4,5]. Despite the paucity of data, child overweight and obesity seem to be rising rapidly in low- and middle-income countries (LMICs) [6] like Vietnam.

In Vietnam, the prevalence of overweight and obesity in children and adolescents is rapidly increasing in urban areas. Within the last decade, there has been a dramatic increase in the prevalence of overweight and obesity in junior high school students in Ho Chi Minh City, the main urban area of the country, from 5.0% and 0.8% in 2002 to 11.7% and 2.0% in 2004, respectively [7]. This upward trend has continued, with the prevalence of overweight and obesity in adolescents increasing from 14.2% to 21.8% over a 5-year period from 2005 to 2009 [8]. Multivariate analyses of survey data collected from 2684 junior high school students found that community and school environments, individual characteristics, and lifestyle behaviors were significantly associated with overweight and obesity in adolescents in Ho Chi Minh City [9].

Recently, a formative study revealed a willingness to increase physical activity to prevent the risk of obesity among junior high school students in Ho Chi Minh City [10]. However, the current physical education curriculum in junior high schools only consists of roughly 2 hours of both theory and practice of sports, which is well below the recommendations of the World Health Organization (WHO) [11].

Systematic reviews have provided evidence that multicomponent interventions have a stronger effect on childhood obesity than programs that focus only on physical activity or dietary behavior change [12,13]. However, there has been no intervention developed in Ho Chi Minh City to promote healthy lifestyles in adolescents. There is currently an urgent need for high-quality, evidence-based programs, which include both nutrition and physical education and a behavior change support system. To design an intervention that combines these components, a pilot study is essential to assess the feasibility and acceptability of the design and delivery strategy of the intervention. Teacher-led or health professional–led interventions can be costly and hard to sustain. In contrast, peer-led programs can be an achievable and effective alternative in economic-constricted settings.

The cost evaluation of the peer-led SALSA (Students As Lifestyle Activists) program in Australian high schools showed that the program was relatively economical to implement [14]. Moreover, the importance of peer relationships and influence intensify during secondary schooling [5,15], and in these settings, peers have a greater influence on the health behaviors of adolescents than parents, teachers, or health professionals [16,17].

The Peer Education and Peer Support (PEPS) project will generate evidence-based insight to design and implement a full-scale intervention to tackle the problem of low physical activity, increased time spent in sedentary activities, and high-energy dense food and imbalanced dietary intake among adolescent in Vietnam and other LMICs.

### Objectives

This paper aims to describe the feasibility and acceptability of the PEPS pilot intervention that combines peer education and peer support delivered in the school setting to promote healthier lifestyle choices among adolescents. Additionally, the impact of the intervention on adolescents’ dietary practices and time spent on physical activity will also be measured.

## Methods

### Study Design

This is a pilot cluster randomized controlled trial in which the intervention combines a peer education and peer support system for a total of 6 months.

### Study Settings

We will select one intervention school in the city center area (District 5) and another intervention school in a suburban location (District 2). Similarly, one control school located in a city center area (District 11) and another control school located further away from the city center (Tan Binh District) will be selected.

### Recruitment

Recruitment of participants for this pilot study include peer educators (undergraduate students at the Pham Ngoc Thach University of Medicine) (PNTUM), peer leaders (grade 8 students), and grade 6 students (the target group).

#### Peer Educators

Undergraduate students at PNTUM will be included as peer educators if (1) they can contribute the time required to work with peer leaders; and (2) they have some skills and experience in programs involving children.

#### Peer Leaders

Students will be recruited as peer leaders if they match the following criteria: (1) they are in grade 8 at the participating schools; (2) they are willing to participate as peer leaders (with parental consent); and (3) they do not have any major medical conditions that may interfere with training and peer-educating activities. All peer leaders are selected based on their willingness to volunteer and by the suggestion of teachers of grade 8 classes. All participating peer leaders will be required to sign a consent form, which is also signed by parents or guardians prior to the next steps.

#### Students

Students will be included in the target group of the intervention if they are: (1) grade 6 students at a participating school; (2) have no major medical issues that may interfere with communication with their peers and learning. Students will be excluded if they meet any of the following criteria: (1) they or their guardian refuse to participate in the intervention; and (2) the student has a medical condition(s) that physically prevents communication and learning. The age range for inclusion is between 10-12 years, and both male and female students will be included.

To increase the participation of schools, we will use a “delayed intervention” technique for the control schools; these schools will be given the opportunity to implement the intervention at the end of the study.

### Intervention Plan

#### Intervention Group

**Figure 1:**
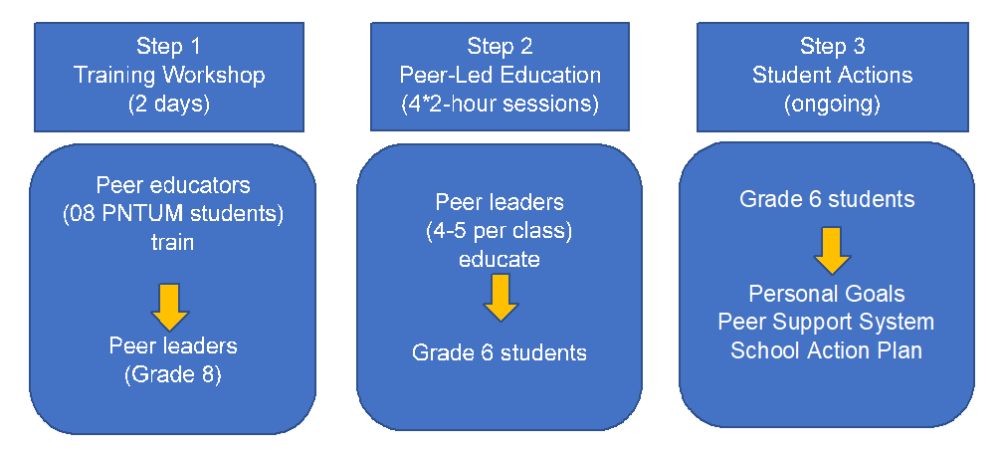
Summary of the Peer Education and Peer Support (PEPS) program. PNTUM: Pham Ngoc Thach University of Medicine.

The peer education part comprises 3 steps that involve training the peer educators (undergraduate students at PNTUM), peer leaders (grade 8 students), and target grade 6 students. Figure 1 illustrates the steps involved in implementing the intervention at a school. Preparations for the program will include explaining the intervention and seeking support with project partners, including the school principal, school staff, and parental groups.

In the first step, two 4-hour training workshops for peer educators will be organized at PNTUM using culturally tailored material for teaching and training (the PEPS Manual). The PEPS Manual will be adapted from the manual of the SALSA program implemented in Australia and other countries [18]. The Salsa program is based on the notions of modeling, self-efficacy, peer pressure, and environment from Bandura’s social cognitive theory and Freire’s empowerment education approach, and aligns with the WHO’s Health Promoting Schools Framework [19,20,21, 22].

We will use the SALSA approach for peer-led education, but the contents will be tailored to the Vietnamese context. We will keep the structure of the education session unchanged (eg, the number of lessons will be three) and broader topics (Food Choices, Movement Matters, and Healthy Lifestyles) will remain unchanged from the SALSA manual. We will, however, change the content and specific examples of food, sport, etc, to make them appropriate to our research setting in Ho Chi Minh City. The module will be initially reviewed by a team of investigators that includes 3 Vietnamese researchers experienced in adolescent obesity research. They will identify content that could potentially require a modification. Then, the module will be shared with the peer educators in a training workshop. Feedback from the peer educators about content modification will be obtained. Finally, we will make suggested adjustments to the content. The SALSA program was successfully implemented in China, Jordan, and Australia. The program had a positive impact on secondary school students and peer leaders in Australia and improved energy balance–related behaviors and intentions to live a healthy lifestyle [14,21,22].

Apart from training on peer education theory and content, the peer educators will be given practical training through time spent with students as they work together as a team to learn about interactive peer education.

The second step is organized by the trained peer educators for peer leaders at the intervention schools and covers the content of the PEPS Manual and approaches to teaching and developing communication skills. The four 2-hour workshops, which take place over the course of 2 weeks, will cover crucial knowledge and updates on healthy, active lifestyles (through 4 lessons of the PEPS Manual) and communication skills for the peer leaders to gain trust and to build strong influence to lead their adolescent peers in this behavior-changing pilot intervention. Interactions between the participants will also be handled based on the principle of transparency, care, and benefits to the student during peer group work. The ratio between peer educators and peer leaders in the training sessions will be maintained as 1:5 to 1:6. During the training, the peer leaders are grouped in working teams of 5-6 peer leaders with 2 peer educators in each team as supervisors. One team is dedicated to a single grade 6 class. The total number of teams equals the total number of grade 6 classes. Prior to the second step, preparations for the program will be made, which include explaining the intervention and seeking support from project partners such as the school principals, school staff, school youth associations, and parental associations.

**Figure 2:**
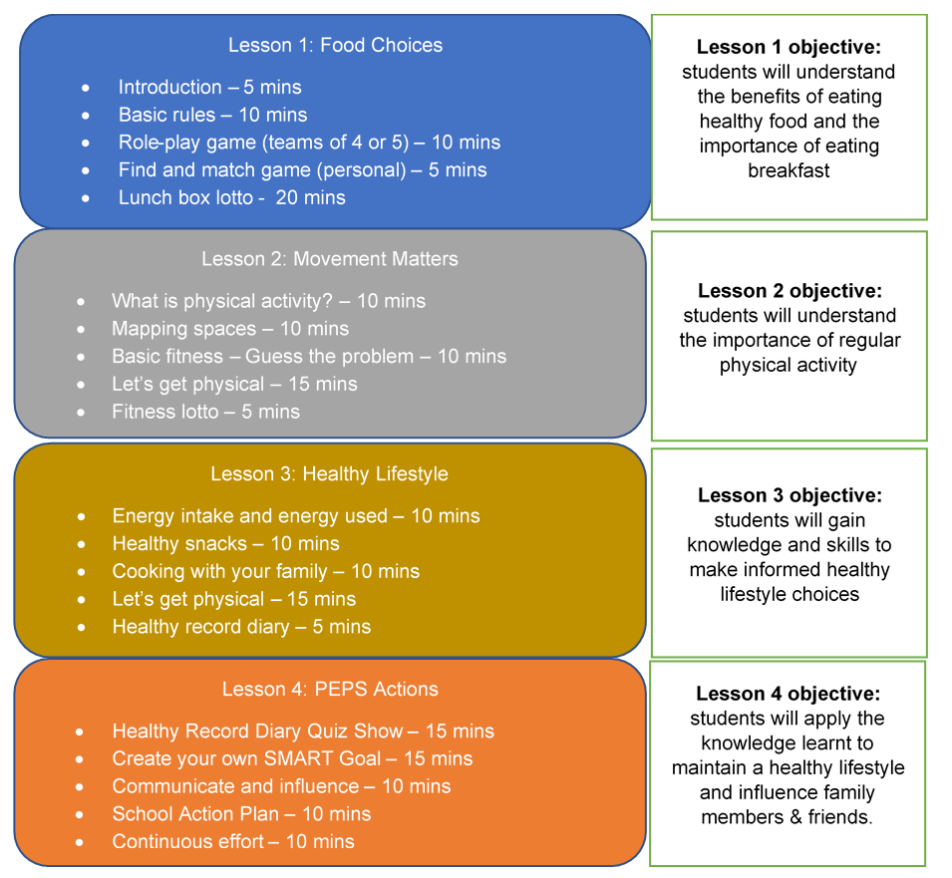
Content of the peer-led education program. SMART: Specific, Measurable, Attainable, Realistic, and Timely.

In the third step, grade 6 classes will participate in four 50-minute teaching sessions following the PEPS Manual, which are carried out by teams of peer leaders. The manual includes the following topics: Food Choices, Movement Matters, Healthy Lifestyles, and PEPS Actions (Figure 2). Each session comprises 2 main parts involving theory and practice skills. The theory part is based on specific facts and active learning that involves student participation and peer-to-peer interaction. Practice skills are designed with team and individual problem-solving games. Each session is delivered once a week by a team of 5 peer leaders and 2 peer educators (as supervisors) to the grade 6 class during the free learning hour every week in the school with consent from teachers and students. The 4 PEPS Manual sessions are delivered in 4 consecutive weeks. If any unforeseen obstacle were to arise and the session of the week is put on hold, the core team members of the PEPS project will discuss with the school principal and school board to find a solution. Preference will be given to reschedule the on-hold session for the next available free time. All sessions will be based on the PEPS Manual. The peer educators will help the peer leaders to practice and deliver the training material in a timely fashion. During any teaching week at any school, there will be one core team member of the PEPS project and one reserve peer educator to deal with logistic issues. During the last peer education session (PEPS Actions), each grade 6 class will produce several strategies and plans. The best plan nominated by the class will be put into practice within the class. The strategies and plans are based on adolescent health benefits, adolescent health objectives, school environment, and the willingness of adolescents to make behavioral changes. Subsequently, two of most suitable plans will be selected to apply to the whole school after the education sessions have finished.

The other part of the PEPS program is a peer-supporting system, which include a behavior reinforcement monitoring system and social network support run and led by peers (peer educators and peer leaders) and monitored by PEPS core team members. The personal reinforcement monitoring system consists of a predistributed personal health record diary, a monthly classroom merit board, and school awards every semester. The class reinforcement system includes organizing school events that stimulate the participation of the class to produce activities for class members. These school events are the plans for action for each school (ie, raising awareness of healthy lifestyles through funny health slogans; welcoming behavioral changes by adopting new action objectives). School events also include sports fairs for all grade 6 classes to take place 1 month after the PEPS teaching sessions end. Online peer support will be introduced to grade 6 students during the teaching sessions (Step 3) to encourage students to view, like, and share health posts on the PEPS Project Facebook public page. An IT (information technology) team working with one author will take responsibility for updating and coordinating the Facebook page. We will also support the schools with a student community/social platform (Facebook, Zalo, ZingVN, or other networks), which will be managed by PNTUM students; teachers will monitor the platform to share experiences, answer questions, and help solve challenges that may arise during school events. Peer educators and peer leaders take on an important role in this system as they serve as online helpers for students as they fulfil the health objectives they devised in session 4 (PEPS Actions). Three core team members will supervise all social networking platforms weekly to help the peer educators deal with students’ queries.

### Control Group

The control group will receive the usual physical education curriculum of the school that comprises three 45-minute sessions (2 required and 1 voluntary) per week. One required session is theory-based and the other two are practical. All sessions are led by physical education teachers.

### Outcome Assessment

#### Primary Outcomes

The primary outcomes include acceptability and feasibility of the intervention to the target population. We will use 3 indicators to measure acceptability: (i) satisfaction, (ii) intent to continue use, and (iii) perceived appropriateness. Feasibility will be assessed by measuring 2 indicators: (i) barriers and facilitators experienced during the implementation of the intervention, and (ii) perceived barriers and facilitators to intervention implementation on a larger scale [23].

#### Secondary Outcomes

Secondary outcomes consist of changes in dietary behaviors, including skipping breakfast, consuming soft drinks and fast food, and fruit and vegetable intake among targeted students (ie, grade 6 students). Dietary intake will be measured by a validated self-administered youth Food Frequency Questionnaire developed and validated in Ho Chi Minh City [24]. This questionnaire, which can be completed in 20 minutes, records the usual frequency of consumption over the past 6 months of 160 foods representing 8 groups: (i) processed foods; (ii) rice, breads, and cereals; (iii) meat, fish, and other seafood; (iv) fruits and vegetables; (v) sweets and snacks; (vi) milk and dairy; (vii) drinks; and (viii) miscellaneous. Information about breakfast and the frequency of out-of-home meals will also be collected.

Secondary outcomes also include time spent in moderate-to-vigorous physical activity as well as time spent performing sedentary behaviors among targeted students (ie, grade 6 students). Level of physical activity is assessed by the Adolescent Physical Activity Recall Questionnaire, which has been validated in Vietnamese adolescents [25]. Activities are assigned a metabolic equivalent score according to the compendium of physical activities. The Adolescent Sedentary Activities Questionnaire, which has good-to-excellent reliability and good face validity in Vietnamese adolescents, is used to measure sedentary behaviors including time spent watching TV, playing games, on the computer, doing homework, and performing sedentary hobbies.

### Sample Size

The sample size for the qualitative assessments (feasibility and acceptability) is based on the principle of reaching data saturation. We will stop data collection and analysis at the point when no significant new data is generated. The quantitative assessments consist of approximately 200 grade 6 students, because we expect small follow-up losses and a likely high correlation between baseline and follow-up outcome measures.

### Intervention Allocation

The assignment of participants to the intervention or control group is based on the location and population size of the school. There are 2 intervention schools and 2 control schools. We allocated the interventions at the school level, but the outcome assessments will be at the individual level.

### Consent

Written informed consent from each participant is obtained. Precautions were taken to ensure participants’ privacy during data analysis.

### Data Collection Methods

#### Qualitative Data Collection

We will assess acceptability by qualitatively measuring how the intended target populations (grade 6 students, peer educator, peer leaders, teachers, and parents) react to the intervention. Feasibility of the intervention will be assessed by qualitatively measuring the reported experience of implementing the intervention by the target populations involved in implementing the project, such as the peer educators, teachers, and project staff. Evaluation of acceptability and feasibility will be performed at the end of the intervention (6 months from the start of the intervention).

We will collect data from purposively selected multiple sources using in-depth interviews, focus group discussions, and direct observation. Qualitative tools, such as in-depth interviews with grade 6 students, peer leaders, teachers, and parents will be used. We will conduct focus group discussions to collect data from peer educators. We will also use direct observation of the school environment and peer leaders’ interactions with the grade 6 students.

During the intervention, we will collect feedback from all participants and institutions after each class. Feedback content will be about the message quality, the instruction of the peer leaders, and the participation of students. Every step is closely monitored and audio or video recorded for analysis. A selection of students will also be invited to qualitative feedback sessions along with meetings with peer leaders weekly in the first month and each month afterwards.

#### Quantitative Data Collection

Dietary behaviors will be assessed using the validated Food Frequency Questionnaire developed for usage among junior high school students and used in previous studies. The secondary outcomes also include time spent on moderate-to-vigorous physical activity and time spent on sedentary behaviors among grade 6 students.

Time spent on moderate-to-vigorous activity will be assessed using the validated Physical Activity Questionnaire, and the time spent on sedentary behaviors will be assessed using the validated Sedentary Questionnaire that were used in previous studies among adolescents in Ho Chi Minh City [26,27].

#### Other Data Collected

Students’ standing height will be measured with a portable direct-reading stadiometer to the nearest 0.5 cm using the standard stretch stature method [28]. Body weight will be measured with shoes and heavy clothes removed using a Tanita electronic scale (Tanita BF 571, Tanita Corporation) to the nearest 0.1 kg. Anthropometric standardization exercises will be conducted by 2 trained data collectors using standard methods to ensure all data will be collected with uniform techniques and standardized measurement procedures [28]. The adolescent’s pubertal status will be self-assessed using a questionnaire with photographs illustrating 5 stages [29] of pubertal development for pubic hair, male genitalia, or female breasts; for female students, the date of their first menstruation is also recorded.

### Data Management

All quantitative data will be collected and entered using tablets at each school.

#### Statistical Methods

Analyses will be conducted using Stata 15 (StataCorp). Descriptive statistics will be used to describe the baseline characteristics of the sample. Feasibility and acceptability will be assessed following the evaluation plan described above. BMI will be calculated as weight in kilograms divided by the square of height in meters (kg/m^2^). The subjects were classified as overweight and obese by applying the age and sex-specific WHO BMI cutoff points [30]. Linear and logistic regression will be used to determine the effect of the intervention on the secondary outcomes, controlling for potential confounders (eg, sex, age, BMI).

### Research Ethics Approval

This study was approved by the Committee of Medical Ethics of the Pham Ngoc Thach University of Medicine (3783/GXN-TÐHYKPNT) and registered with the Australian New Zealand Clinical Trials Registry (ACTRN12619000421134).

## Results

The first participant was enrolled in early September 2018. Recruitment was completed by March 2019. We delivered the peer education intervention at the start of each school year over 3 months for all new grade 6 adolescents in the allocated schools, followed by peer support and home engagement activities over 6 months until the end of the school year. However, the outcome assessments will be based on a cohort of adolescents only. There was a baseline assessment and 2 post-intervention assessments: the first immediately after the intervention to assess the short-term impact and the second at the end of the school year to assess the sustained impact on changes in adiposity, diet, and physical activity. In total, 326 grade 6 students were recruited. Data collection was completed in September 2019.

## Discussion

This paper presents the protocol for a pilot trial to determine the feasibility and applicability of a peer education and peer support intervention to promote healthy lifestyles for the prevention of overweight and obesity in adolescents in Ho Chi Minh City. The intervention combines a peer education and peer support system for a 6-month period.

A recent systematic review of peer-led interventions to prevent child and adolescent obesity found that peer-led interventions significantly impacted both BMI and combined BMI outcomes of children and adolescents [31]. The review examined 25 peer-reviewed papers from 14 studies with 2506 participants and found a mean estimate of the effect on BMI to be a reduction of 0.15 kg/m^2^ (95% CI –0.26 to –0.03). Subgroup analyses revealed an estimated effect twice as strong in adolescents aged 11 years or older than with younger children. The impact of the intervention was also found to be much stronger for longer-term interventions of 6 months and more. However, the studies in the review were conducted in high-income countries and many had small size samples. Findings from the review highlight the need for well-designed studies in LMICs with enough power to more precisely assess the impact of peer education and to evaluate the best approach to peer leading. Additionally, in many school-based obesity prevention trials, the initial positive impact was greatly reduced or eventually neutralized once the teachers or health workers were no longer onsite.

Hence, this study will generate evidence of feasible and acceptable approaches to interventions to prevent excess weight gain and methods of peer-led education to promote healthy lifestyles to prevent obesity in adolescents in LMICs. The findings of the study will be used to inform the development of a larger scale cluster randomized controlled trial to examine the impact of an intensive, multicomponent, school- and home-based lifestyle promotion intervention using innovative peer education methods to improve diet and physical activity and reduce obesity in urban Vietnamese adolescents.

## Abbreviations

DALY: disability-adjusted life year
IT: information technology
LMIC: low- and middle-income country
PEPS: Peer Education and Peer Support
PNTUM: Pham Ngoc Thach University of Medicine
SALSA: Students As Lifestyle Activists
WHO: World Health Organization
